# Exploring Effects of Information Filtering With a VR Interface for Multi-Robot Supervision

**DOI:** 10.3389/frobt.2021.692180

**Published:** 2021-09-21

**Authors:** Daniel Butters, Emil T. Jonasson, Vijay M. Pawar

**Affiliations:** ^1^ Autonomous Manufacturing Laboratory, Department of Computer Science, University College London, London, United Kingdom; ^2^ Remote Applications in Challenging Environments (RACE), UK Atomic Energy Authority, Culham Science Centre, Abingdon, United Kingdom

**Keywords:** virtual reality, human-robot interaction, multi-robot systems, interface design, robot supervision, information display, user study, information filtering

## Abstract

Supervising and controlling remote robot systems currently requires many specialised operators to have knowledge of the internal state of the system in addition to the environment. For applications such as remote maintenance of future nuclear fusion reactors, the number of robots (and hence supervisors) required to maintain or decommission a facility is too large to be financially feasible. To address this issue, this work explores the idea of intelligently filtering information so that a single user can supervise multiple robots safely. We gathered feedback from participants using five methods for teleoperating a semi-autonomous multi-robot system via Virtual Reality (VR). We present a novel 3D interaction method to filter the displayed information to allow the user to read information from the environment without being overwhelmed. The novelty of the interface design is the link between Semantic and Spatial filtering and the hierarchical information contained within the multi robot system. We conducted a user study including a cohort of expert robot teleoperators comparing these methods; highlighting the significant effects of 3D interface design on the performance and perceived workload of a user teleoperating many robot agents in complex environments. The results from this experiment and subjective user feedback will inform future investigations that build upon this initial work.

## 1 Introduction

The main goal of this experiment is to explore how information filtering strategies affect the user experience and performance of a single person supervising and controlling multiple robots. Supervising and controlling robots requires transmission of information between the robot system and the human operator through an interface. One difficulty with this setup is that a multi-robot system is very complex, and may overwhelm the supervisor with too much information. A potential solution to this issue is to filter the information so that the operator can see a subset of information at a given time without being overwhelmed. This experiment aims to explore novel information filtering strategies that link spatial and semantic information filtering to the information within a multi-robot system. The operator is able to control and view information through a 3D Virtual Reality interface. As the scale and complexity of engineering structures around the world increase, the need for multiple robots carrying out complex maintenance or decommissioning tasks is growing. In many use cases such as nuclear, space, and disaster recovery, it is not practical for humans to enter these operational areas in person due to physical inaccessibility, environmental hazards, or simply due to the large monetary and temporal cost of training, employing and equipping humans for these tasks. As a result, these use cases require remote robotic systems which can range from fully teleoperated to fully autonomous. Traditional fusion remote maintenance systems such as the one which can be found at JET, the Joint European Torus^1^ involves a person-in-the-loop telerobotic manipulator with Haptic feedback, called the MASCOT (Hamilton et al., 1995; Murcutt et al., 2011). Whilst capable and adaptable, this type of maintenance system suffers from the fundamental limitation of requiring one person to operate the manipulator and several more people in the supporting team, operating some of the functionality of the MASCOT as well as supporting equipment (Boessenkool et al., 2014). This is in part due to the fundamental human limitation of how much information any one person can perceive and process at any one time, as well as the lack of built-in assistive low-level control of the MASCOT.

Supervision and control of multiple robots in a remote maintenance and inspection task creates requirements for the interface. The task involves supporting robots to move large, extremely heavy objects. For example, replacing lithium breeding blankets (12.5 m long, 80 tonnes) requires positioning of ±10 mm accuracy (Buckingham and Loving, 2016). This leads to the requirement that structures must be inspected with high accuracy to avoid neutron leakage. An assessment of remote maintenance requirements for DEMO (DEMOnstration Power Plant) determined that priority should be given to designing maintenance systems to be as quick as possible as the cost of electricity is inversely proportional to the availability of the reactor (Loving et al., 2014; Ward et al., 2001).

The future success of alternative cleaner sources of power generation such as Nuclear Fusion will depend heavily on the price-performance of commercial power stations. In turn, the price-performance of Fusion is heavily dependent on the efficiency and capability of the remote maintenance systems (Petkov et al., 2020). Maintenance operations usually require shutting down the reactor. The duration of reactor shut down needs to be minimised to maintain the financial feasibility of the reactor (Petkov et al., 2020). Maintenance carried out in hot cells will need to use multi-robot systems instead of single robot systems to minimise the time taken for maintenance tasks (Farquhar et al., 2019). Historically, the limitations of these legacy maintenance systems have simply been tolerated due to the limitations of practical technological solutions, however, the development of completely remotely maintained hot-cells required for future reactors such as ITER, EU-DEMO or STEP indicates a need for the development of disruptive methods and technologies to simplify these challenges.

Hence, one method for future fusion experiments and commercial reactors to improve their commercial viability would be to reduce the number of operators required to operate the maintenance equipment through automation of certain tasks, and to improve the methods of presenting information to the remaining operators/supervisors. A promising way to reduce the reliance on human operators is to increase the autonomy of the robotic system. The use cases of nuclear, space and disaster recovery have high requirements for safety due to the potential risk to human life and monetary cost of malfunctions. Current state of the art fully autonomous robots cannot operate safely in all situations and environments. Multiple surveys (Kunze et al., 2018; Guiochet et al., 2017; Wong et al., 2018) describe the current and future technical challenges that need to be solved before autonomous systems can guarantee that they will meet all safety requirements without human intervention. Therefore, even in the case of a fully autonomous robotic system, there is a need for some level of human supervision to satisfy the necessary safety requirements.

Human supervision of multi-robot system requires an interface that shows information regarding the robots and environment. This information is complex and needs to be displayed in such a way that it conveys the information without overwhelming the supervisor. One way to make sure that information is accessible without overwhelming the user is filtering the information. Robot system information can be viewed as a hierarchy, with the environment being the highest level, the team of robots as a level lower, the individual robot functions below that and the joints of each robot below that. A potential way to display this information is through linking semantic and spatial filtering to the scope of information. Semantic filtering can be applied to situations where the information can be separated into distinct types. These types range from internal robot information such as robot joint values, to the high level function of a robot, to the path through the environment that they will take. Spatial filtering can be applied to situations where the information is linked to physical or virtual objects that are situated within a 2D or 3D environment. This can apply to individual robots and other objects within the environment. Spatial filtering is designed to be intuitive and closer to the real world, as the operator sees more information from close objects than distant objects.

**FIGURE 1:**
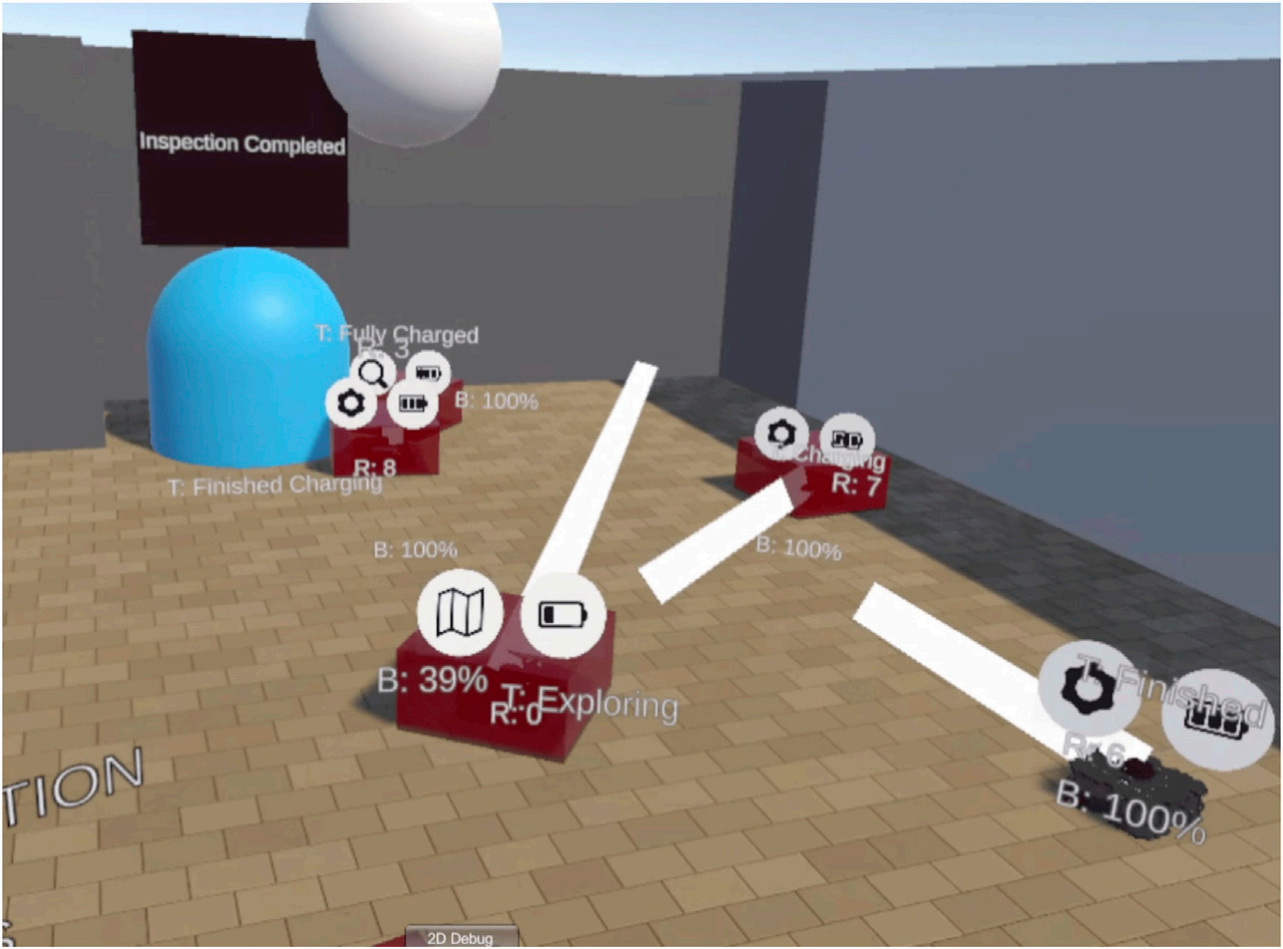
A representative example of the interface. Three layers of information can be seen here: internal text labels (e.g. task status), banner icons (robot category, battery level) and path plans (white lines representing the planned motion of the robot).

This study aims to explore and investigate spatial and semantic information filtering methods that could aid the teleoperation and supervision of multiple robots in complex environments. In doing so, we will find out whether information filtering can affect task performance or affect the cognitive demands of tasks. Any improvements to task performance or cognitive load could improve the capabilities of supervisory interfaces for multi-robot systems, and potentially reduce the requirement for supervisor staff in future facilities such as the EU-DEMO fusion project. Figure 1 shows a representative image of the interface as seen by the user in Virtual Reality. This experiment uses Virtual Reality as the display and input method primarily because VR can offer new ways to display information that could be advantageous.

To compare these methods, we conducted a user study with 26 participants to assess their potential for supervising and directing multiple robots to perform a series of cognitively demanding tasks. In these tasks, nine robots with different roles (Explorer, Support, and Inspector) are directed around a semi-known environment. Explorer robots can explore unknown areas of the environment, Support robots can charge/repair other robots, and Inspector robots can inspect objects in the environment. The user must direct the robots to explore the environment for any robots/hazards, repair any broken robots and inspect all objects within the environment. The tasks in this experiment are designed to be similar to the use case of operators supervising multiple semi-autonomous robots in order to perform maintenance in an inaccessible environment, such as areas surrounding a fusion reactor. Participants reported that the interface was easy to use and could be applied to a more demanding task. The significant findings from the user study include that the spatial filtering of information was disfavoured by users and semantic filtering produced no significant difference to the baseline case. In addition, valuable knowledge was gained regarding the experimental design needed to evaluate the performance of these new disruptive interfaces such as VR. The main change needed for future experiments will be to design tasks such that users are overwhelmed with information in the case with no filtering. We conclude with a discussion about the trade-offs in each design and the future areas that can build upon this work.

## 2 Related Work

### 2.1 Interface Design for Complex Systems

Ecological interface design (Vicente and Rasmussen, 1992) is a framework for human-machine interface design based on cognitive processing being separated into Skills, Rules and Knowledge. The framework aims to enable the operator to complete tasks without requiring a higher level of cognitive processing than the task demands. Sawaragi et al. investigated the ecological interface design framework for the use case of robot teleoperation (Sawaragi et al., 2000). They found that a shared 3D virtual space that displayed obstacles around a robot allowed “naturalistic collaboration between a human and a robot’s autonomy at the skill level”. Anokhin et al. proposed an ecological interface design (Anokhin et al., 2018) for monitoring the processes of a nuclear power plant. Their findings were that the ecological interface reduced the error rate, reaction time and operator load. The ecological interface framework is an example of information being divided in a hierarchy of the level of reasoning required to deal with it.

Hedeyati et al. investigated the use of AR for display of sensor information close to the real location of the robot (Hedayati et al., 2018). The authors experimented with superimposed camera feeds and other information for teleoperation of a UAV. They conclude that the interface design improved performance over existing systems, as their system does not require users to divide attention between the robot and a separate camera feed. This experiment has informed the decision to superimpose labels upon robots within our own investigation.

### 2.2 Information Filtering and Clustering

3D interfaces where the user is able to move around can use a combination of spatial and semantic filtering, as shown in Julier et al., 2002. The authors present a method for spatially filtering data with AR. Objects have a region of interest, with more interesting objects having a larger region. When a user enters the region of interest, information about the object is displayed. This method has potential applications in robot supervision, but may not offer enough fine control to access relevant information quickly for this use case. T. Butkiewicz investigated the design principle for an AR system to aid in marine navigation (Butkiewicz, 2017). The authors used spatial filtering to fade out the marine navigation overlay elements based on both their importance and their distance from the user.

Clustering of information can help to avoid visual clutter when displaying a set of information in AR. Tatzgern et al. propose a method for clustering information labels in AR. Individual pieces of information are leaf nodes in a tree structure (Tatzgern et al., 2016). An algorithm clusters information based on their similarity or spacial proximity and displays a level of detail corresponding to the amount of space in the field of view of the AR. This method could be useful for robotic supervision, however, it is not yet clear how it could be applied to types of information which are not aggregated or averaged easily, such as task status or robot sensor data.

### 2.3 View Management

View management is a method for maximising legibility (the degree to which text can be read easily) and information visibility (the degree to which information can be seen easily) when displaying a set of information labels in 2D or 3D interfaces. Madsen et al. proposed a method for view management which uses 3D geometric constraints to maintain legibility and temporal coherence for labels attached to objects (Madsen et al., 2016). This allows labels to move in one dimension along a 3D pole constraint attached to the subject of the label. This could be used for textual or pictorial labels in the context of multi-robot supervision, but is not suitable for all types of information being displayed, and does not account for cases where the density of information causes labels to overlap.

A 2D force based view management technique is proposed in (Hartmann et al., 2004). This uses attraction to the object being labelled, repulsion from the edge of the object, repulsion from other labels, repulsion from the edge of the display and repulsion from all other elements to dynamically place labels in a legible manner. This is a temporally coherent way to determine label placement, and a similar method has been extended to 3 dimensions in this paper.

### 2.4 Multi-Scale Virtual Environments

Multi-Scale Virtual Environments (MSVEs) are useful for displaying any type of hierarchical data. Hierarchical data has multiple levels of information, with smaller levels residing inside larger ones. For example, galaxies contain solar systems, solar systems contain planets and so on. MSVEs have been used in VR experiences in order to educate about subjects such as the human body (Bacim et al., 2009) and objects in space (Deyang and Norman, 1994). They have also been evaluated in the context of human-human interaction through a giant and miniature person collaboration in VR (Piumsomboon et al., 2019).

Kouril et al. proposed a method to apply labels to multi-scale environments where there are multiple instances of the same object (Kouril et al., 2019). They apply labels to groups of objects instead of single objects if it is appropriate to do so (clustering) and implement a strategy to only label representative instances of objects in order to reduce clutter. This strategy takes advantage of the fact that objects in the environment are repeated and only need representative labels. In robot supervision, information types are often similar across different robots. However, it is not always suitable to only show representative examples because a particular piece of information could be needed from any of the robots being supervised. The concept of hierarchical information display is applied in this paper to the levels of scope for each layer of information.

### 2.5 Virtual Reality for Teleoperation

Since VR technology has increased in fidelity and reduced in cost over recent years, it has been investigated as a medium for interfaces for robot supervision and control. Jang et al. use VR for virtual kinesthetic teaching of a robot arm (Jang et al., 2019), (Jang et al., 2021) and discuss the advantages of a system that integrates ROS, Unity and Virtual Reality. Whitney et al. describe an interface for connecting two ROS controlled robot arms in VR and remotely operating them with sensor data (Whitney et al., 2018). The authors demonstrate that it is possible to display high fidelity sensor data in VR from ROS and use it to operate a dual arm robot. This type of interface has potential to be extended to a system with larger numbers of robots. High fidelity sensor data is outside the scope of our work but is planned to be investigated in future works.

Four variants of a multi-robot interface were proposed and evaluated in (Roldan et al., 2017). These interfaces included a conventional 2D interface, a VR interface, and predictive versions of these interfaces. They found that the VR interface improves the operator situational awareness without increasing workload, and the predictive interface did not significantly affect the operators. The scope of the Rodan et al. study did not include interfaces where there are more than three robots or the effect of information filtering on user perception and performance.

## 3 Design Requirements

Design requirements are highlighted in bold in this section. For the anticipated use case of remote maintenance of a fusion reactor, the human supervisor is there to direct robots and prevent any actions that may be undesirable. In this future scenario, the robots will not be directly teleoperated, but directed to complete a task autonomously, with human interventions and verification when necessary. To support this, the supervisor should be able to quickly read information from the interface to support quick decision making. Therefore, the interface should be easy to read and understand. Additionally, time delay should be minimised.

The amount of information that fully describes a multi-robot system can be very large. Any type of information could be needed, ranging from the sensor readings, to the state of the internal operating system, to the interactions between robots, to information about the surrounding environment. It is possible for a large amount of information to overwhelm users when the density of information presented to them is too high. Azuma et al. explain this data density problem in the context of Augmented Reality (AR) (Azuma et al., 2001). To prevent the user from being overwhelmed, the information management system should limit the amount of information exposed to the supervisor at a given time.

Finally, supervisors may need to make decisions based on their general situational awareness of the environment and objects within it. Therefore all information contained within the environment must be accessible by the user through the interface.

Classical methods for information management in AR when representing data in an egocentric view of the environment include Spatial and Semantic filtering (Julier et al., 2002). Spatial filtering uses the distance from the user’s eye to a given augmented reality label’s location to determine which information is displayed. Information density can be changed manually or autonomously by adjusting the cut off distances for each piece of information. Spatial filtering of information is selective rendering of information based on the assumption that the importance of the subject relates to the physical distance between the subject and the observer. When this assumption holds true, this type of filtering can remove irrelevant information, allowing useful information to be more easily ingested by the observer.

Semantic filtering uses the type of information to choose whether to display information or not. Information density can be adjusted through manipulating which types of information are displayed at a given time. Semantic filtering traditionally requires users to manually select the information that should be displayed, which can slow down the process of accessing relevant information. Semantic filtering of information is selective rendering based on the assumption that the importance of the information relates to the type. View management is a process where information is moved around to maximise legibility. This approach can help with legibility but does not limit the amount of information exposed to a user at a given time. In this work, view management is used in all experiments to aid legibility. Semantic and Spatial filtering are evaluated separately and in combination with each other.

## 4 Objectives

This study will investigate variations of Semantic and Spatial filtering that are linked to the layers of information about the multi-robot system. Both the type of information displayed and the distance at which information is displayed on each layer can be coupled to the scale of the user. The information related to each object will be located close to the object in a 3D environment. This allows users to search physically through an environment for an object, mimicking how people search through real 3D environments, instead of finding the relevant information in a more traditional 2D interface. Information about an object is located close to or in the same position as the object, which is a more natural way to view information than referring to a separate database of information.

The information contained in the state of a multi-robot system and environment can be divided into different levels. Each layer of information can be classified as one of three levels:

• Individual—The information pertains to a single robot. For example, the battery level of a robot or the type of robot.
• Multiple—The information pertains to multiple robots. For example, the planned trajectory of a robot that could cross the path of another robot, or a set of robots that are grouped together.
• Overview—The information pertains to the entire environment. For example, the density of robots in a given area, or areas in the environment that are contaminated.

A virtual reality headset was chosen as the medium for this interface to be built around. VR is an appropriate interface (with potential to be disruptive to the field) because it is immersive, gives depth cues through stereo display, and enables intuitive input systems to be designed. VR places the user within a 3D environment, which allows them to search for information based on its 3D position or proximity. VR also has the capability of combining traditional 2D UI elements and 3D environment elements to richly render information.

We believe the proposed interface design is a novel way to supervise and control multiple robots. The interface extends information filtering techniques by coupling them to the low, medium and high level information relevant to a robot system. The 3D interface implements intuitive gestures in order to both control how the user is receiving information and how the user responds to it. This interface design takes advantage of the fact that information about robotic systems and their environment are inherently associated with a given location, so the user can search through data by directly navigating within the virtual environment. We will evaluate the potential of this interface and the different levels of information filtering in this work.

## 5 Interface Design

### 5.1 System Overview

**FIGURE 2:**
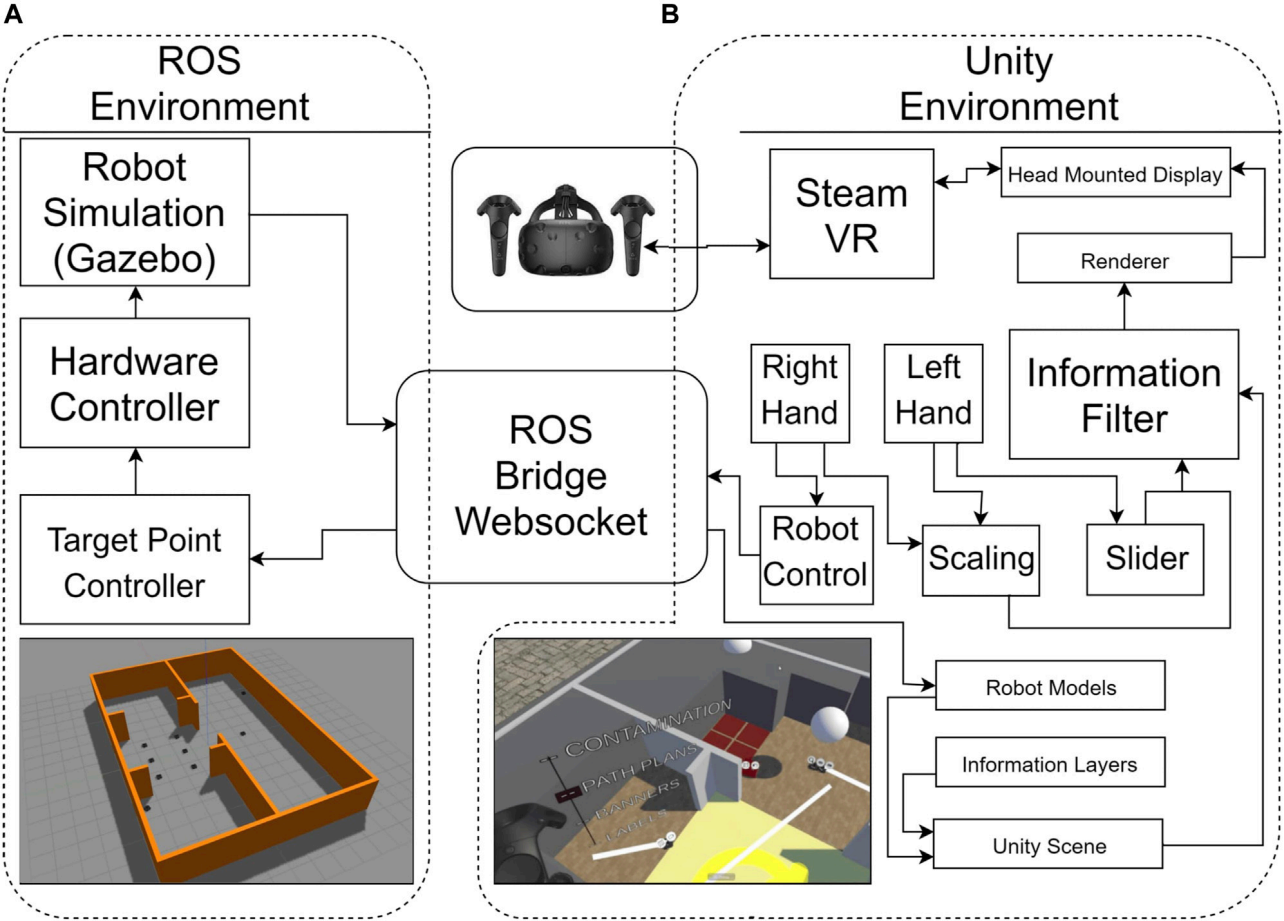
An overview of the setup that contains the ROS simulation (Ubuntu, **A**) and the Unity VR program (Windows, **B**). The communication between the computers used the Rosbridge Websocket.

Figure 2 shows the System Overview for this experiment. There is a VR environment running in Unity connected over a websocket to a ROS simulation of the robots. The simulated robots are small non-holonomic ground robots called Turtlebot3. These robots are well integrated with ROS, both for simulations and hardware, allowing for the same information to be transferred in either case. In a real world remote maintenance and inspection task, it is likely that larger, heavier duty AGV (automated guided vehicle) robots with similar motion protocols would be used. For each task, nine robots are simulated within the same environment using the Gazebo simulator, an open source software that is packaged with ROS. This software is capable of simulating hard body collisions, friction, sensors and robot dynamics, enabling the same control signal to be sent to a simulated or real robot, with the interface receiving the same types of sensor feedback. In many future scenarios such as nuclear maintenance, purpose-built facilities are being specifically designed to support accurate localisation for robotic maintenance systems within a well defined environment. Jonasson et al. (2021) compare three key current technologies for their effectiveness in robot localization in nuclear facilities. For this experiment it is assumed that the underlying structure of the environment and robot positions are accurately known, though the Inspector bot is used to validate this information and reveal any differences between the pre-known environment model and the actual situation.

**FIGURE 3:**
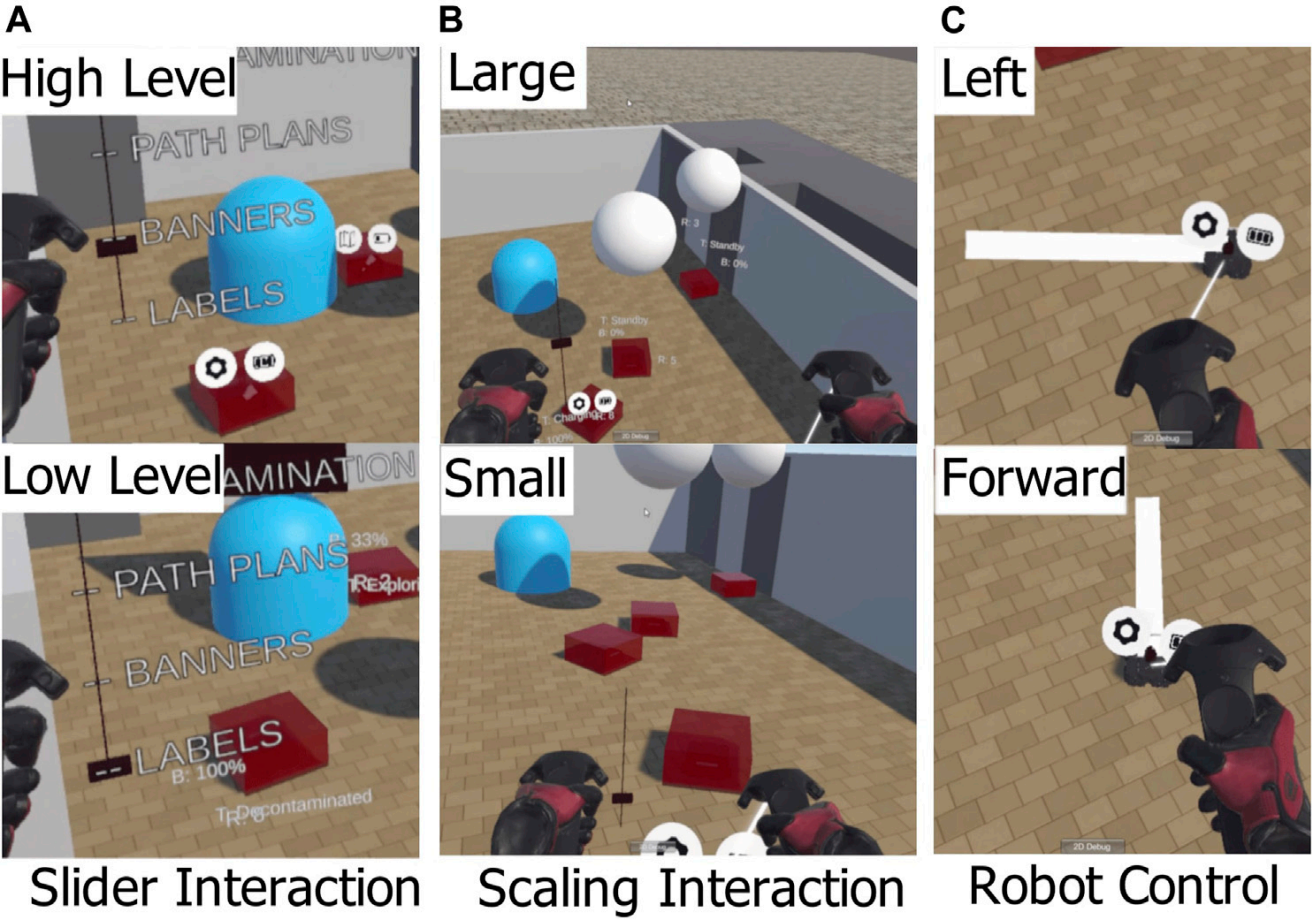
The four interaction methods used to control robots and manipulate the information displayed. The slider method **(A)** is used for the interface variant V2. The scaling method **(B)** is used for V3, V4 and V5. All variants use the same method to command the robots to move **(C)**.

The communication between the simulation in ROS and the VR system used the Rosbridge Websocket (Mace et al., 2014)). This allows systems outside of ROS to send and receive messages using JSON serialisation. In Unity, the JSON messages are interpreted using the Siemens ROS-Sharp package (Bischoff, 2017). The simulated robots use two wheel differential drive to control angle (yaw) and speed. For this experiment, the control of each robot was simplified to a point to point controller. When the controller is supplied a target pose, the robot turns on the spot to face towards that position, moves forward until it is within a 2.5 cm radius of that position and stops. All robot simulation and control was carried out in ROS Kinetic (Quigley, 2009). A ROS node receives messages from Unity when the user instructs a robot to move. The VR headset used was the HTC Vive (Niehorster et al., 2017) and the 3D interface was built using the Unity game engine (Bartneck et al., 2015). The robot models were displayed in Unity with the same position as in the simulation. The user can set the target position of robots, move around the environment, interact with a slider and change the scale of the user relative to the environment. These capabilities are shown in Figure 3. To virtually move around the environment, the user can press a side button and move the controller. The user’s perspective is translated along the vector of the difference between the controller position and the position when the button was initially pressed. This allows the user to move around the environment without physically moving around using their feet.

To change the scale of the user relative to the environment, the user could hold a side button down on each controller and perform a pinching gesture. This gesture made the user bigger if they moved their hands apart, and smaller if they moved their hands closer together. This gesture was designed to be a 3D analog to the “pinch to zoom” gesture that is common in touch screen interfaces. When a user changes scale, the ground plane of the user is kept the same, so the user increases in height relative to the ground plane. This interaction method is shown in Figure 3B.

The user selects a robot to control by moving close to the robot. The robot closest to the user’s right hand is then selected. When the user holds down the right trigger button, the target position of the robot is mapped to the position of the right hand relative to the position when the trigger was originally pressed. When the user releases the trigger, the target position is set and the robot attempts to move to that position. This interaction method is shown in Figure 3C.

### 5.2 Information Layers

The layers of information used in this experiment are used to provide a complex set of information for the participant to interact with. In order to access information relevant to the task, the participant must use the interface to change the filtering of information.

Internal robot information (referred to as L1) includes robot number (each robot is assigned a unique number), task status (textual and numerical data) and battery percentage. This layer is comprised of textual information and could include other types of information if desired. Information for L1 is stored within the Unity (C#) scripts that implement the VR interface, and is relayed directly to the user. Elements in this layer are displayed in Unity using a TextMeshPro object. Each label has its rotation set to face the player camera and its position is set by the result of three forces. The first force attracts the label towards the robot that it is labelling. The second force repels labels away from each other. The third force attracts the labels to a plane that is perpendicular to the direction of the player camera. The resulting 3D force is applied to each text label attached to the robots in order to minimise overlapping and maximise legibility of labels. This layer is shown in Figure 5 (L1).The second layer (referred to as L2) is a set of two icons that are attached to a point above each robot. These icons display battery status (low, medium or high level of charge) and robot category. Robots can be one of three categories: Explorer, Support and Inspector. This layer is shown in Figure 5 (L2). Information within L2 is stored within C# scripts that run the experiment.

Explorer robots are able to explore the environment, looking for obstacles and other robots. In many practical maintenance, decommissioning or disaster recovery situations, this “exploration” is really a verification that the environment observed by the robot is unchanged from an initial digital model or as-built drawings. For example, future nuclear fusion facilities will have accurate digital models of the environment, which the robots can autonomously verify by observing the environment directly.

**FIGURE 4:**
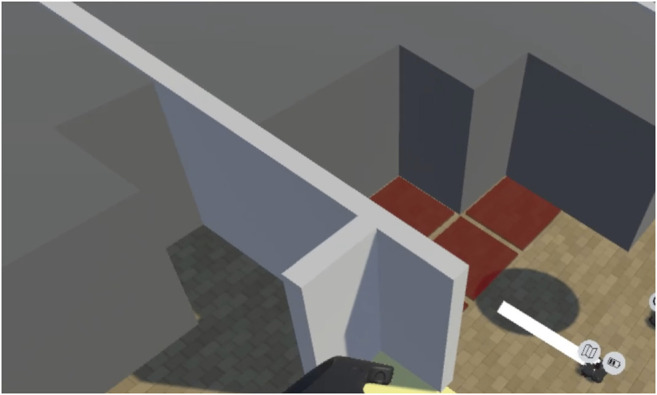
“Fog of war” is displayed as the dark grey opaque blocks. These areas represent the parts of the map that have not been verified by an Explorer robot.

The state of exploration is represented in the Virtual Environment as a “fog of war” type system. Opaque grey blocks obscure regions of the environment that have not been verified by an Explorer robot. This is shown in Figure 4. When an Explorer robot moves to within 1.5 m of an unexplored region, the region is marked as explored. This means that the opaque block disappears, allowing that part of the environment to be seen by the operator. The explored portions of the map are updated through a C# script in the Unity environment. This system enforces the idea that, while the state of the environment is known to some degree at the start of the work being carried out, it must be verified before any robots can safely operate in that area.

Support robots are able to charge the battery of other robots. When a user moves a Support robot to within 0.5 m of another robot, it charges their battery at a constant rate. The charge of a robot battery is displayed on the internal labels or the battery icon above a robot. The task status of the Support robots will change from “Charging” to “Finished Charging” when the other robot has been fully charged. In every task in the experiment, at least three robots will start off with zero percent battery and will need to be charged. This charging robot is a simplification of expected recovery tasks that might need to take place in future robotic maintenance deployment scenarios.

Inspector robots are able to inspect objects in the environment. In every task, there are two objects that need to be inspected. A user can view the inspection progress by looking at the internal label of the object that is being inspected. The Inspector robot’s task status will also change from “Inspecting” to “Finished Inspecting” when the inspection has been completed.

**FIGURE 5:**
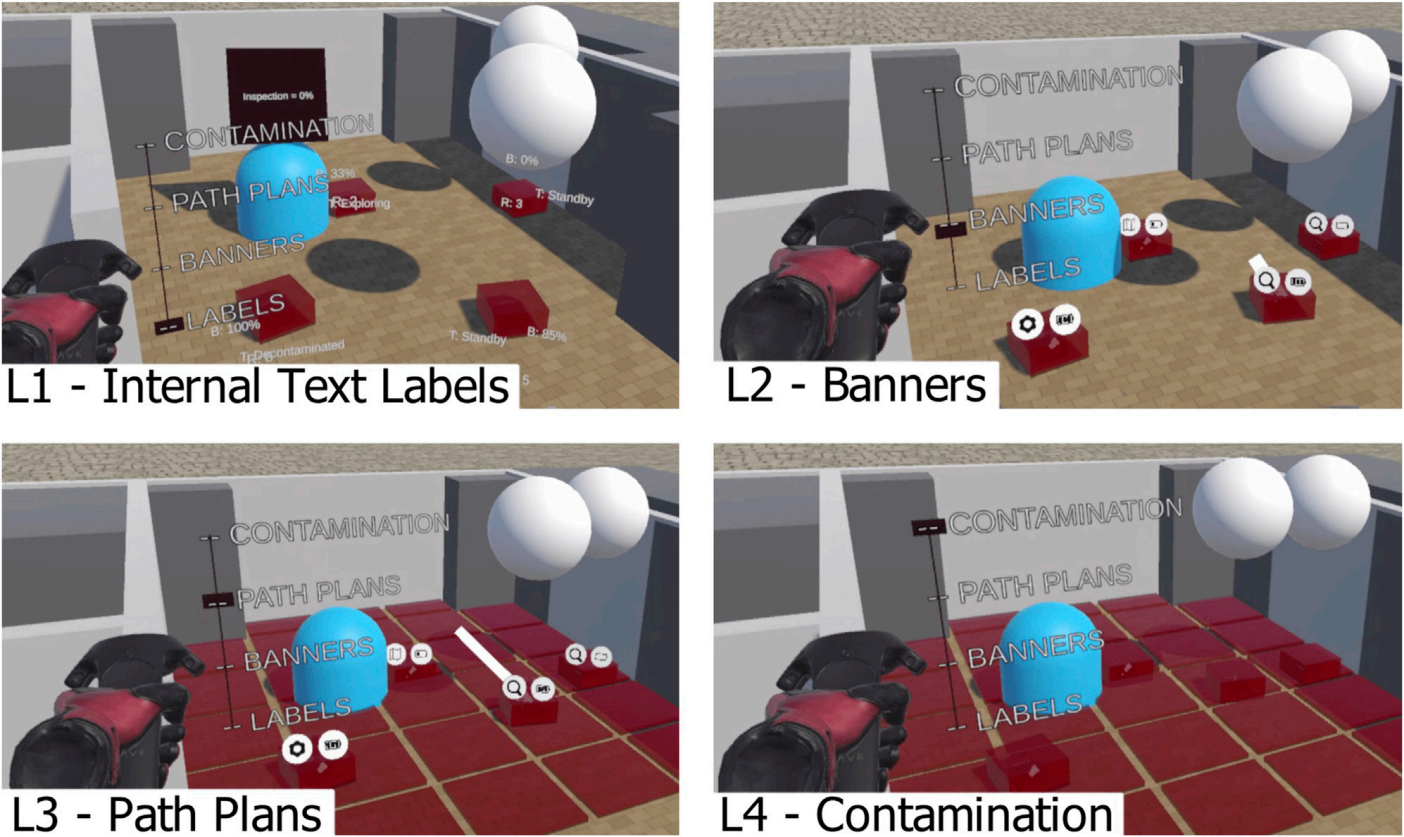
The four layers of information displayed. L1 is textual labels displaying internal information about the robot (e.g. task being performed). L2 has banners displaying external information about the robot (e.g. type of robot). L3 displays lines that represent the planned paths of each robot. L4 displays “contaminated” areas within the environment.

The third layer (referred to as L3) displays the planned path of each robot. It is displayed as a line which extends from the robot to the target position. This allows users to plan robot motions that will not interfere with the motion of another robot. This layer is shown in Figure 5 (L3).

The fourth layer (referred to as L4) displays the “contamination” of the environment. In this study, it is possible for a robot to become contaminated by entering a contaminated area, and decontaminated by entering the decontamination zone. This layer displays contaminated areas with a transparent red square. This layer is shown in Figure 5 (L4). In many nuclear applications, areas of the facility can have contaminated material (radioactive or chemical) in them, so anything leaving those areas (including robotic maintenance equipment) needs to be decontaminated or cleaned before safe removal is possible. In these experiments, a simplified implementation of this provides more complexity and information for the user to deal with.

### 5.3 Interface Variants

There are five variants of the interface and each participant tried all variants in a randomised order. The participant was able to scale themselves up or down in every variant.

• V1 is the Baseline variant. All information is displayed and unfiltered.
• V2 is the Slider variant. This allows the user to control the Semantic filtering (toggling layers on or off) using a slider on their left hand (shown in Figure 3A).
• V3 changes the Semantic filtering based on the scale of the user. This allows the user to control the Semantic filtering by changing their scale (small scale corresponds to L1, large scale corresponds to L4).
• V4 changes the Spatial filtering based on the scale of the user (small scale corresponds to only seeing information close to the user, large scale corresponds to also seeing far away information).
• V5 uses both Semantic and Spatial filtering controlled by the scale of the user.

All variants including the baseline case (V1) were in virtual reality as the focus of this work was on the effect of the information filtering when coupled with the scale of the user. Task performance with a 2D display variant would not be comparable because the task would have to be adapted to a mouse and keyboard interface. Changing the scale of the user in a 2D interface would have different effects to virtual reality as the depth cues in a stereoscopic display inform the user of their size, and in a 2D interface these depth cues are not there. In addition, the scope of the work is limited to the effect of these information filtering methods within virtual reality. Comparisons between VR and 2D interfaces have been investigated for robot teleoperation (Lager and Topp, 2019; Roldan et al., 2017).

### 5.4 Filtering of Information

In this study, there are two ways used to filter information and two input methods for controlling them. Semantic filtering is implemented by toggling layers on and off in order to only display a subset of layers. The toggles are linked to either the scale of the user, or the user controlled slider. When the scale (or slider) is small, the “Individual” information layers are shown. As the user (or slider value) gets larger, the “Multiple” level information is shown and then the “Overview” level information.

Spatial filtering is achieved by modifying the draw distance of components in each layer. This means that if the object is further away from the player camera than the maximum draw distance, it is not rendered. The draw distance for each layer is linked to the scale of the user or the manually controlled slider.

To control the filtering, a conventional input method can be used. This uses a gesture where the user holds down the left trigger button and can move their hand up or down. This method is shown in Figure 3A. A slider displays their corresponding position on a line. This slider is used in one experiment variant (V2) to control the semantic filter by mapping the layer selections to the position on the slider.

Another input method for controlling the filters is to couple the filtering to the scale of the user. In this case, the scale of the user relative to “life size” scale is used to input a value to the filtering system. The scaling gesture is shown in Figure 3B. A side button on each controller is held down, then the user can move their hands apart to get bigger or move their hands closer to get smaller.

## 6 Experiment Design

The user study had 26 participants (19 male and 7 female) with ages ranging from 21–54 and a mean age of 30.04 (with standard deviation of 9.20). This user study was approved by the UCL Ethics Committee, Project ID 13305/002. Half of the participants were professionals involved with robots, with varying levels of expert knowledge of teleoperating the MASCOT system described in the introduction. Seven participants were students involved with robotics and six were professionals not involved with robotics. Each participant trial took around 45 min to complete.

For each variant of the interface evaluated in the experiment, the same task is set for the user. The user must 1) Use the Explorer robots to find all three Inspector robots that need charging. 2) Use Support robots to charge three Inspector robots. 3) Use the Inspector robots to inspect two objects. 4) Decontaminate any robot that has become contaminated by moving these robots to the start area.

### 6.1 Experiment Procedure

At the start of the experiment, each participant was given an introductory page of information that told them what the work is about and the motivations behind the work (including completion of a consent form if they decide to do the experiment). Participants were then given a tutorial on the controls for changing scale, controlling robots and using the slider. The tutorial made sure that the participants could use the controls, and set a minimum skill level for the experiment. The tutorial consisted of going through each subtask that is required for the main task. For the main task, there were three Inspector robots in the environment that need to be found and have their batteries charged by Support robots. These robots change position randomly between trials to reduce learning effects. A robot that needs to be rescued could potentially be in any pose within its own workspace. These robots are in unknown positions within the environment to increase the difficulty of the task. In addition to this, in the anticipated use case of remote maintenance of a fusion reactor, it is possible for the localisation of a robot to fail, in which case it cannot be assumed that the failed robot’s position is known.

Each repeat of the scenario has two objects that need to be inspected by Inspector robots. These objects stay in the same position over all experimental trials, and their positions are initially shown to the participant in the tutorial. This was based on the idea of a remote maintenance task with static objects that need to be inspected routinely. Each participant completed the task five times, with the variants presented in a randomised order. The set of interface variants are described in the Interface Design section. After each repeat, the user filled out a survey described in the next subsection.

At the end of the experiment, the user was asked to rank all of the interface variants, and asked open ended questions as a prompt for qualitative feedback.

### 6.2 Measurements

All participants were asked to provide initial information about their age, gender, professional background, familiarity with robotics and familiarity with VR. To measure the perceived difficulty of the task with each variant, the NASA TLX survey (Hart and Staveland, 1988) was filled out after each task. This survey measures the mental demand, physical demand, temporal demand, overall performance, effort and frustration perceived by the user when completing the task. Three additional factors were also rated on a 7 point rating scale: “How easy was it to control which information was displayed?“, “How easy was it to find relevant information?” and “How easy was it to understand the information?“. For these questions, an answer can range from 1 to 7, representing “Very Easy” and “Very Hard” respectively. At the end of the experiment, the participant was asked to give feedback from an open ended prompt: “Please provide any comments you have about the different interfaces”. They were also asked to rank the five interfaces, with 1 being the best and 5 being the worst.

The performance of the participant in each task was recorded. This metric was derived from the components of each task that they managed to complete, and the time taken to do so. Each task was stopped after 240 s, unless the task was completed sooner. The participants were informed that performance was measured by how many of the tasks they completed in the time given. The performance metrics were: (A) Number of Robots Charged (0–3), (B) Number of Objects Inspected (0–2), (C) Number of Robots Contaminated (0–9), (D) Time Taken (seconds). P (performance) was then calculated using this formula:

$$P=\frac{100}{120}*\left(20*\left(A+B\right)-10*C+20*\frac{240-D}{240}\right)$$

Performance is formulated so that the value is greater when more subtasks have been completed, and smaller when more robots have been “contaminated”. The performance value increases if the tasks are all completed before the cut off time of 240 s.

## 7 Results

### 7.1 NASA TLX and User Feedback

**FIGURE 6:**
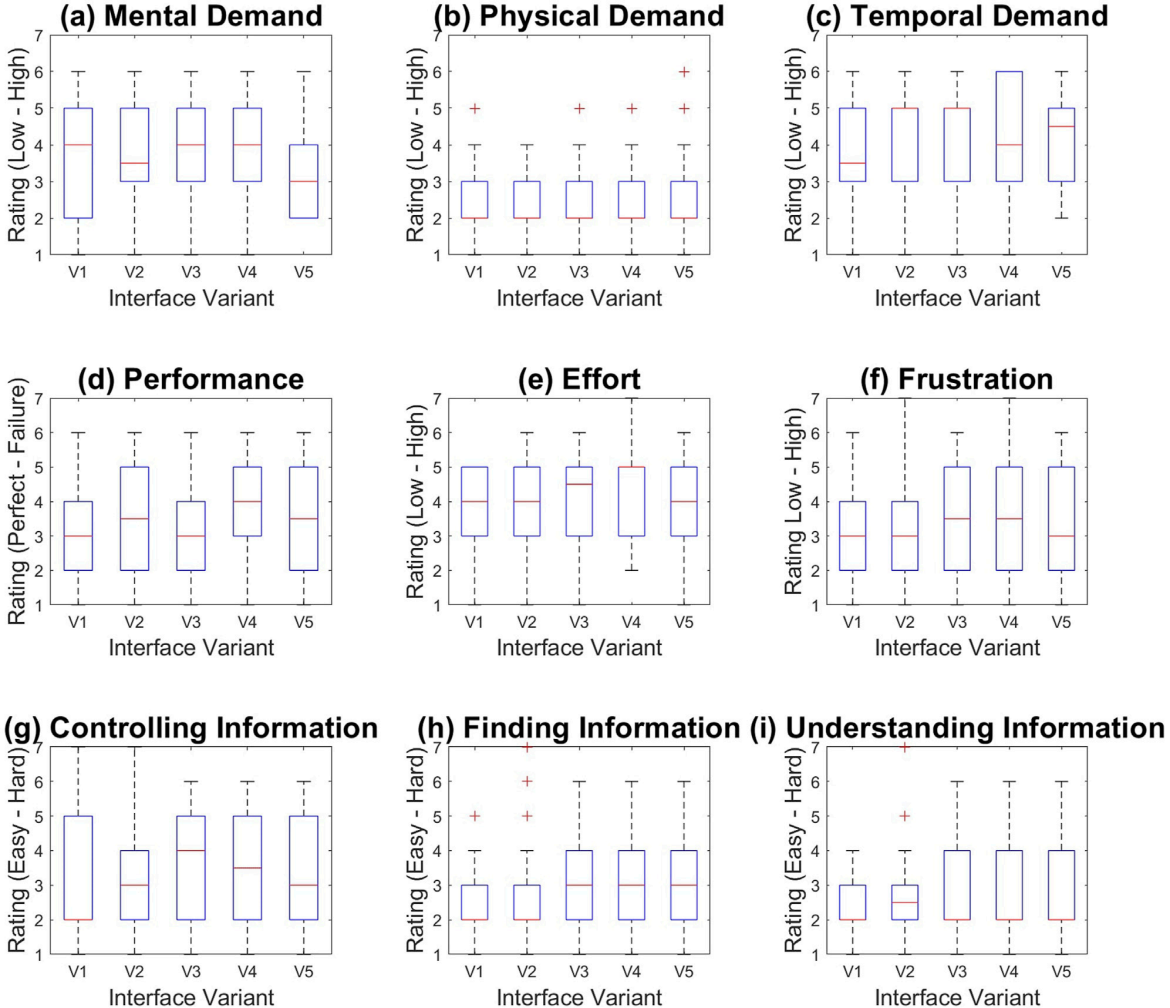
Box and whisker graphs showing user feedback for the Interface Variants (V1–V5) over 9 factors **(A–I)**.

Figure 6 shows box and whisker graphs for the 9 factors users rated between 1 and 7. We performed ANOVA one way statistical analysis (significant threshold = 0.05) on all factors in the experiment, with the independent variable being the variant of the interface (V1–V5). One-way ANOVA determined no significant difference between groups [F (4,125) = 2.1692, *p* = 0.0763] for the “Finding Information” category (shown in Figure 6H), however a Tukey post hoc test (*p* = 0.0399) revealed that V1 (2.3462 ± 1.1981) was significantly rated better than V5 (3.4231 ± 1.3616). The effect size corrected for sample size was Cohen’s d = 1.4827. Users found it significantly easier to find information with Interface Variant V1 (where all information was available to them independent of scale, and no filtering occurred) than with Interface Variant V5 (where Semantic filtering and Spatial filtering were linked to the scale of the user). This indicated that the combination of spatial and semantic filtering made the act of finding information be perceived as more difficult than in the baseline case. Other factors had no significant effects between interfaces.

### 7.2 Performance and Interface Rankings

**FIGURE 7:**
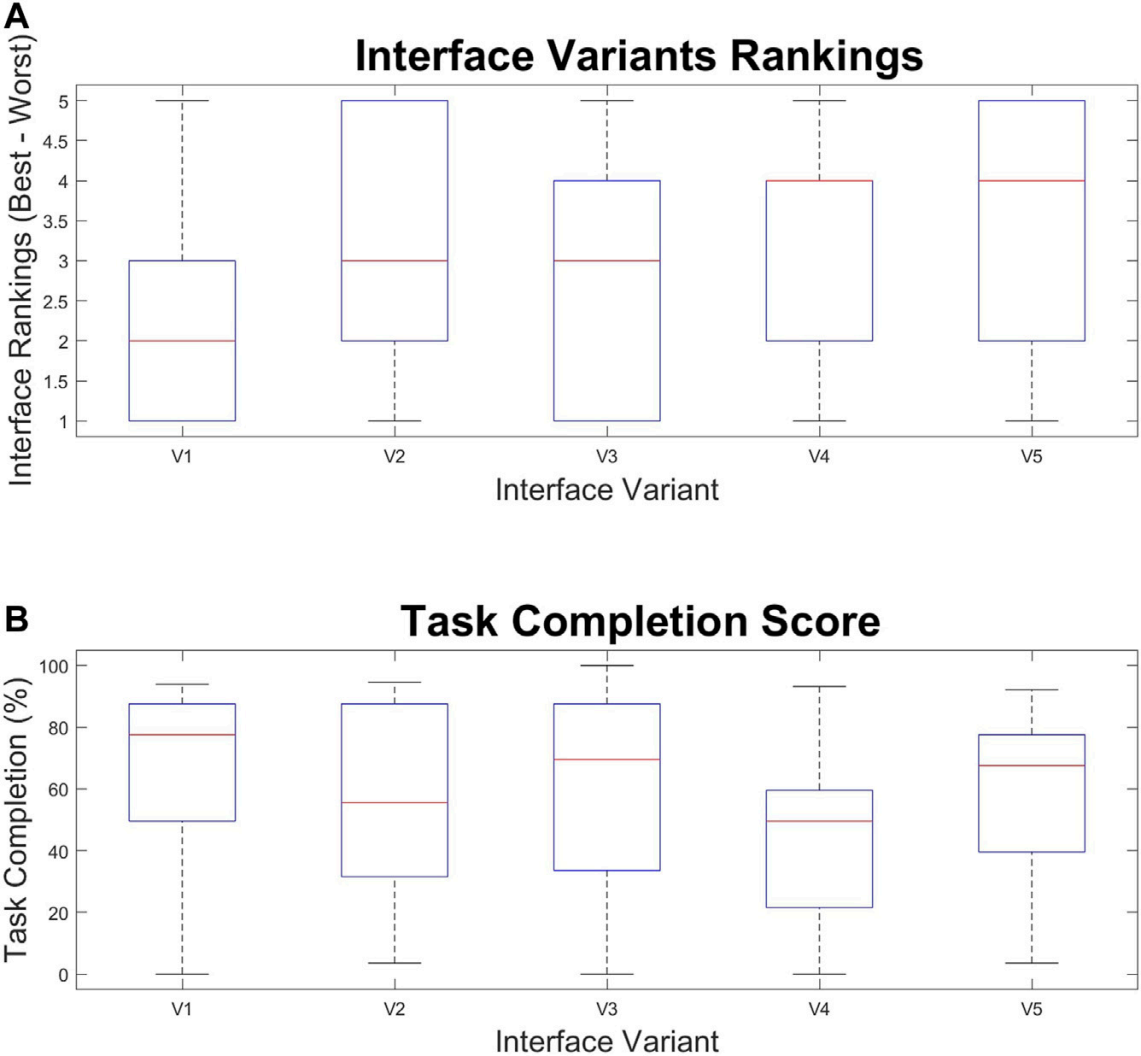
Box and whisker graphs showing **(A)** User rankings of each interface (1 is best, 5 is worst) and **(B)** Task completion percentage over the Interface Variants (V1–V5).

After completing all of the tasks, the participants were asked to rank the interfaces from 1 to 5, with 1 being the best. A box and whisker plot of these rankings are shown in Figure 7A. The participants also had their completion of each task measured for each interface variant. This metric corresponds to how much of the task the participant was able to complete and time for completion. The formula to determine this metric is explained in the Experiment Design section. A box and whisker plot for this data is shown in Figure 7B.

Task performance for each interface and interface rankings were evaluated with ANOVA One Way analysis. This analysis found two significant effects on the rankings of the interfaces, finding a significant difference between groups [F (4,125) = 3.3018, *p* = 0.0131]. When comparing all of the interfaces, variant V1 (2.2308 ± 1.3945) was ranked significantly better than V2 (3.3077 ± 1.4905) with Tukey post hoc test = 0.0374 and corrected Cohen’s d = 1.6395. V1 was also ranked significantly better than V5 (3.4231 ± 1.5013 with Tukey post hoc test *p* = 0.0149 and corrected Cohen’s d = 1.7524. This indicated that the slider input, and the combination of spatial and semantic filtering were not preferred when compared to the baseline case. This could indicate that users prefer information filtering coupled to scale rather than using the manual control of the slider. These results may also suggest a preference for interfaces where less information is hidden from the user.

Variants with spatial filtering (V4, V5) were compared to variants without spatial filtering (V1, V2, V3) and ANOVA One way determined that the groups were significantly different [F (1,128) = 4.7286, *p* = 0.0315]. Variants without spatial filtering (2.7692 ± 1.5290) were ranked significantly better than variants with spatial filtering (3.1519 ± 1.3213) with Tukey post hoc test *p* = 0.0297 and Cohen’s d = 1.2139. This result indicates that the participants perceived that the spatial filtering hindered them when completing tasks with this interface. There were no significant differences in task completion found within the data.

## 8 Discussion

After completing the experiment, participants were asked to submit written feedback about the interfaces and the experiment in general. Topics that were mentioned most frequently within the comments were noted. Not all participants commented on all aspects of the experiment, so the total number of comments about a topic vary. Each comment was then analysed to determine whether the participant expresses positive or negative feelings about it. Table 1 shows this data.

**TABLE 1:** Topics that were most commonly talked about in written feedback from participants. Each comment was determined by the authors to be positive or negative about a subject. The numbers determined to be positive/negative are shown here. Not all topics were mentioned by all participants, so the total number of comments for each topic varies.

Topic	Majority like/agree with the topic	Proportion that like/agree with topic
Seeing as much information as possible at the same time	Yes	10 out of 11
Scaling as a method of changing information	Mixed	5 out of 9
Interfaces where fewer actions are needed to access information	Yes	9 out of 9
Spatial filtering	No	1 out of 5
Using the slider as a method for controlling information	Mixed	3 out of 5
The interface was easy to use or understand	Yes	4 out of 5
The symbols used in the experiment were easy to understand	Yes	4 out of 4
The interface could be effective for more complex scenarios	Yes	3 out of 3
A more customisable interface	Yes	3 out of 3

The qualitative survey results can provide some insight into the preferences of people using these types of interface. These preferences may be used as guidelines for future design work.

One aim of this study was to determine if the initial interface design was easy to use for the task of multi-robot control and supervision. Participants in general found the interfaces easy to use, with 16 out of 26 participants able to complete the entire task within the time limit and 4 out of 5 comments saying that the interface was easy to use. Participant P8 commented: “The interfaces as a group were very easy to use and to understand what information was being displayed”.

The quantitative results indicated that the main factor that affected user experience was the difficulty of finding information. The data suggests that the combination of Spatial and Semantic filtering increased the diffiulty. For this variant (V5), in order to access the internal text labels, the user would also have to be close to the label they want to access in order to read it. This means that two commands (scaling and moving) are required to access this information, whereas the other interface variants only require one. The participants expressed a strong preference for fewer actions to access information, with 9 out of 9 comments supporting this idea. For example: P17 “Spatial meant that a large amount of movement of the virtual camera was required to keep track of the status of the various robots. This meant that a quick survey wasn’t possible”.

The rankings of each interface suggested that the spatial filter in particular is not preferred by the users, with 4 out of 5 comments expressing negative feelings towards spacial filtering. One user commented: P16 “The range limit was unhelpful for the icons as it prevented me from getting a wide-scale view though I may have preferred it for the text.” This could suggest that a different filtering approach can be used for different modes of displaying information such as symbols and text information.

3 out of 5 comments indicated positive feelings about the slider input method, so there is no clear consensus. An example of a positive comment: P14 “I preferred the slider version as it meant i didn’t have to change the overall view i.e. I didn’t have to make myself bigger in order to see it.” However, others preferred controlling information by changing the scale of the user P16 “I actually found the semantic task easiest as it forced me to zoom in rather than keeping a bird’s-eye view which actually made some of the task feel more manageable.”

Having explored the initial feasibility of the interface, the effects of information filtering and input methods (the main goal of this work), the results have highlighted some future directions to take this research further. Firstly, some users reported difficulty with selecting which robot to control: P13 “Selecting individual robots could be tricky if they were close together and relied on a lot more user activity to get the right one”. This could be improved with a finer control system which allows users to scroll through robots based on their proximity.

Users have also suggested that the Slider input method is useful when they want to see different information while maintaining the same viewpoint, and the Scaling input method is useful when they want to focus on a particular robot. These comments suggest that individual users may prefer different input methods for different situations, and could lead to future investigations into customisable interfaces that can be modified as the situation demands. This is also supported by the 3 out of 3 comments that suggested positive opinions of a future customisable interface.

Another result from this work is that the users did not find the amount of information presented even in the V1 interface to be enough to require finer filtering. This is supported by the comments, with 10 out of 11 saying that they preferred seeing as much information as possible at once. One participant commented: P18 “I think that the slider control would be more suited to tasks more complicated than this test where it is useful to filter out information.” This suggests that this interface has not reached the limit for the amount of information it could display, and could be applied to a more demanding task, with greater amounts of information. This more challenging scenario would be likely to yield clearer preferences for differing types of filtering. The methods explored in this work can be further investigated as potential solutions to the problems anticipated within the areas of future fusion reactors, including maintenance, nuclear decommissioning, disaster recovery and space operations, allowing fewer supervisors to supervise greater numbers of robots.

Additional strategies for controlling the information could be evaluated such as clustering information, filtering based on relevance to the task and machine learning to predict what information is needed at a given time. Future work could also evaluate the performance of this design when compared to a more traditional 2D interface used to control multiple robots. Another avenue to explore would be ways to filter high fidelity sensor data as well as the textual and numerical information shown here. Sensor data such as laser scans and point clouds are inherently multi dimensional, and so will need new strategies for filtering of information.

## 9 Conclusion

In this study, a novel virtual reality interface to supervise and control multiple semi-autonomous robots during a remote maintenance and inspection task was developed. In order to meet task requirements, we proposed a novel design for an interface where users were able to scale themselves relative to the environment, while supervising and controlling multiple robots to complete a complex task. The novel interface design linked the spatial and semantic filtering of information to the hierarchical information within the multi-robot system. We have verified the initial feasibility of this design and explored how information filtering can affect the user experience. We also compared interaction methods for controlling the information in the environment. The results from this experiment indicate that in the particular scenario modelled, the filtering of information did not significantly improve the user experience or performance, and that spatial filtering was experienced as being detrimental. However, this is likely to be caused by the information provided to the users not being sufficient to overwhelm them. Due to this factor, showing all information at once was the most beneficial since it did not require the users to take any action to see further information. This result indicates that future experimental designs should ensure that the tasks are demanding enough to create a difficult challenge for the participants in the base case. In this experiment, the challenges could have been adapted to be more difficult to overcome. Despite this, we gathered useful user feedback which will influence the design of future interfaces and experiments. Future work will build upon the initial exploration in this study by measuring the effects of information filtering in tasks with greater information density. Additional works will include comparing this interface to a more traditional 2D interface, and exploring additional strategies to improve interaction with the robots and the display of information.

## Data Availability

The raw data supporting the conclusion of this article will be made available by the authors, without undue reservation.

## Footnotes

1 JET is operated and maintained by the United Kingdom Atomic Energy Authority (UKAEA) on behalf of the EUROfusion consortium and located at the Culham Science Centre in Oxfordshire, United Kingdom
